# A novel behavioral paradigm reveals the nature of confidence computation in multi-alternative perceptual decision making

**DOI:** 10.21203/rs.3.rs-5510856/v1

**Published:** 2024-12-11

**Authors:** Kai Xue, Medha Shekhar, Dobromir Rahnev

**Affiliations:** School of Psychology, Georgia Institute of Technology, Atlanta, GA

**Keywords:** Confidence, metacognition, perceptual decision making, computational models

## Abstract

A central goal of research in perceptual decision making is to determine the internal computations underlying choice and confidence in complex, multi-alternative tasks. However, revealing these computations requires knowledge of the internal representation upon which the computations operate. Unfortunately, it is unknown how traditional stimuli (e.g., Gabor patches and random dot motion) are represented internally, which calls into question the computations inferred when using such stimuli. Here we develop a new behavioral paradigm where subjects discriminate the dominant color in a cloud of differently colored dots. Critically, we show that the internal representation for these stimuli can be described with a simple, one-parameter equation and that a single free parameter can explain multi-alternative data for up to 12 different conditions. Further, we use this paradigm to test three popular theories: that confidence reflects (1) the probability of being correct, (2) only choice-congruent (i.e., positive) evidence, or (3) the evidence difference between the highest and the second-highest signal.The predictions of the first two theories were falsified in two experiments involving either six or 12 conditions with three choices each. We found that the data were best explained by a model where confidence is based on the difference of the two alternatives with the largest evidence. These results establish a new paradigm in which a single parameter can be used to determine the internal representation for an unlimited number of multi-alternative conditions and challenge two prominent theories of confidence computation.

## Introduction

Humans possess the metacognitive ability to assess the accuracy of their own decision through confidence ratings (Fleming, 2024; Mamassian, 2020; Rahnev, 2021). Accurate metacognition is important in many domains, such as learning and the ability to engage in sequential decisions (Aguilar-Lleyda et al., 2020; Hainguerlot et al., 2018; Schunk & Ertmer, 2000). A critical goal in the field is identifying the computational mechanisms behind confidence in complex, multi-alternative tasks (Rahnev et al., 2022). However, a key challenge to accomplishing this goal lies in unraveling how external sensory information is transformed into internal evidence representations. The reason this transformation is critical is that all theories of confidence are built on assumed internal representations, but it is often difficult or impossible to independently confirm whether the assumed internal representations match the actual ones (Miyoshi et al., 2024; Shekhar & Rahnev, 2024b).

There have been many controversies in the field that can be traced back to the inability to determine the exact nature of the internal representations evoked by sensory stimuli. For example, Rahnev et al. (2011) proposed a mechanism that attention influences the internal distribution by reducing the variance of the signals. However, while this model was able to explain several counter-intuitive findings, it was criticized by researchers who showed that alternative combinations of internal activations and decisional strategies can also explain the observed behavioral results (Denison et al., 2018; Lee et al., 2023). Similarly, Odegaard et al. (2018) proposed an “inflation” model to argue that humans overestimate perceptual capacity within their visual periphery, but the model has been criticized on the grounds that different assumptions about how sensory stimuli produce internal activations are possible (Abid, 2019).

Uncertainty about the nature of internal representations is common because it is not known exactly how standard manipulations for traditional stimuli – such as contrast manipulations for Gabor patches and coherence manipulations for random dot kinematograms – affect the internal evidence distributions in multi-alternative tasks. For example, simple 2-choice tasks are typically modeled using signal detection theory (Green & Swets, 1966), which assumes that each stimulus category gives rise to a Gaussian distribution of internal evidence. However, stimulus manipulations such as contrast or coherence may change the means (of one or both distributions) or variance of the distributions (or both), and it is often impossible to know a priori how to model such manipulations (Shekhar & Rahnev, 2024b). Further, the lack of direct mapping from manipulations to internal activation effects sometimes necessitates that each level of contrast or coherence is modeled using its own free parameter(s) (Shekhar & Rahnev, 2024a), resulting in overparameterized models prone to overfitting. The difficulty of mapping sensory stimuli to internal activations becomes even greater for multi-alternative tasks, which explains why such tasks are rarely used in perceptual decision-making studies.

Here we introduce a novel behavioral paradigm based on a dot numerosity task which enables the mapping of a potentially unlimited number of conditions to corresponding internal activations using as little as a single free parameter, even for multi-alternative tasks. We demonstrate that a one-parameter decision model can explain the data from 3-choice tasks in two experiments with up to 12 conditions. We further use these data and the model linking sensory stimuli to internal activations to test three theories of confidence computation. We find strong evidence against the notion that confidence is based exclusively on decision-congruent evidence, with our results being best explained using a model that postulates confidence based on the difference in evidence between the highest and second highest sensory activation. Overall, our findings establish a paradigm in which a single parameter can describe the internal representation of a broad array of multi-alternative conditions and allow us to begin unraveling the confidence computations used in multi-alternative tasks.

## Results

**Figure 1.**
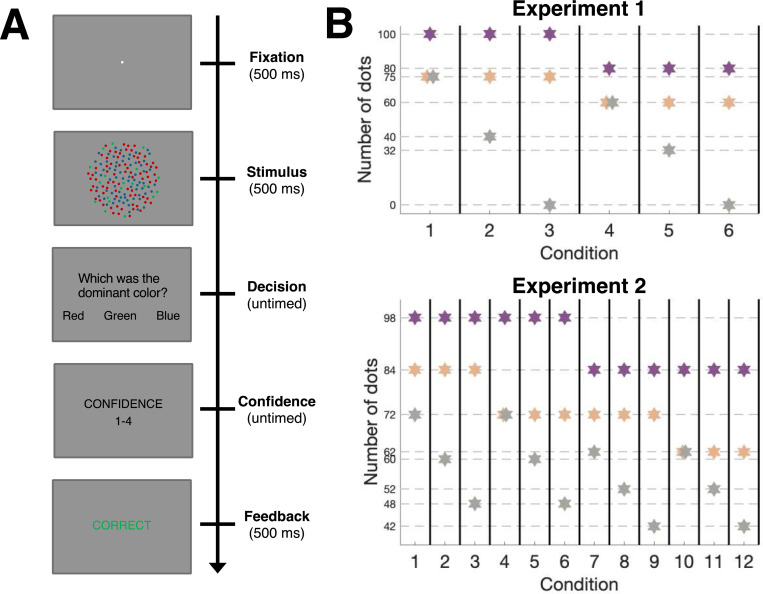
Experimental design. (A) Trial structure. On each trial, we presented a cloud of dots of three different colors. Subjects first indicated the dominant color (i.e., the color with the largest number of dots) and then rated their confidence on a 4-point scale. They received trial-by-trial feedback in both experiments. (B) Number of dots in each condition. Experiment 1 (top) included six conditions. In conditions 1–3, the highest dot number was always 100, the second highest was always 75, and the lowest changed across conditions to be 75, 40, and 0. In conditions 4–6, all dot numbers were 80% of the ones in conditions 1–3. Experiment 2 (bottom) included 12 conditions. In conditions 1–6, the highest dot number was always 98, while the second highest was 84 in conditions 1–3 and 72 in conditions 4–6. The lowest dot numbers took the values 72, 60, and 48 for both conditions 1–3 and 4–6. In conditions 7–12, all dot numbers were about 85.7% of the ones in conditions 1–6, such that the highest number was always 84, the second highest was 72 or 62, and the lowest was 62, 52, or 42. The purple, orange, and gray stars represent the colors with the highest, second highest, and lowest dots numbers.

We conducted two experiments in which subjects completed a dot numerosity task where they judged the dominant color in a cloud of dots with different colors (Figure 1A). Both experiments featured 3-choice tasks (choosing between red, green, and blue) and subjects provided confidence on a 4-point scale after each response. We included six conditions in Experiment 1 and 12 conditions in Experiment 2 (Figure 1B). All conditions featured one color with the highest number of dots (i.e., the dominant color), and most conditions additionally featured one color with the second-highest dot number and lowest dot number (two conditions from each experiment had an equal number of dots for the two non-dominant colors). Across all conditions in both experiments, we counterbalanced the color arrangement so that each color (red, green, blue) appeared an equal number of times with the highest, second-highest, or lowest number of dots. The experiments were designed to examine several behavioral effects, focusing on how overall dot numerosity and the relative proportion of dots between colors influence decision-making and confidence.

### Behavioral results

**Figure 2.**
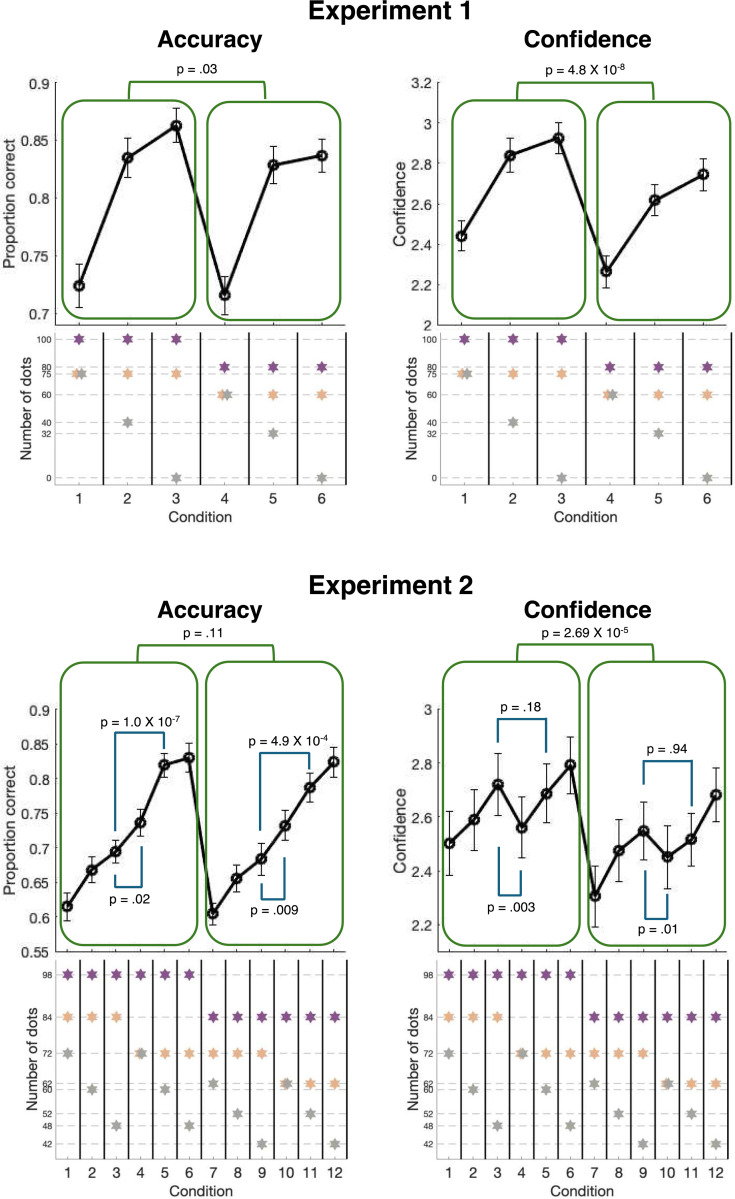
Accuracy and confidence for each condition in Experiments 1 and 2. The left two panels display accuracy data, and the right two panels display confidence data. The top two panels depict results from Experiment 1, and the bottom two panels depict results from Experiment 2. The number of dots in each condition is at the bottom of each figure. Green boxes represent the Numerosity effect, while the blue lines represent the adjacent option effect. Error bars represent SEM.

Before fitting any models, we examined the qualitative patterns in the accuracy and confidence data (Figure 2). In Experiment 1, both accuracy and confidence increased as the number of dots from the least frequent color decreased (Accuracy: t(24) = −11.02, p = 7.13 X 10^−11^, Cohen’s d = −2.20; Confidence: t(24) = −8.85, p = 5.06 X 10^−9^, Cohen’s d = −1.77). Similarly, in Experiment 2, accuracy and confidence generally increased as the number of dots from the two least frequent colors decreased (Accuracy: second choice: t(14) = −16.73, p = 1.19 X 10^−10^, Cohen’s d = −4.32, third choice: t(14) = −9.82, p = 1.17 X 10^−7^, Cohen’s d = −2.54; Confidence: second choice: t(14) = −4.95, p = 2.14 X 10^−4^, Cohen’s d = −1.28, third choice: t(14) = −7.89, p = 1.62 X 10^−6^, Cohen’s d = − 2.04). Beyond these commonsensical results, we also observed two important qualitative patterns that can serve as critical targets for modeling. First, increasing all dot numbers by a constant ratio had almost no influence on accuracy but robustly increased confidence. We call this the “Numerosity effect”.

Second, in Experiment 2, we observed an effect related to the relative numbers of dots for the two non-dominant colors (i.e., the second and third most frequent ones). Specifically, in several pairs of conditions, we manipulated the dot numbers such that the distribution between the two non-dominant options became more or less even, while keeping the dominant color’s dot count constant. This manipulation created a trade-off between the two non-dominant options, which manifested in robust confidence-accuracy dissociations: conditions with a more even distribution between the non-dominant options tended to yield higher accuracy but either unchanged or even decreased confidence. We refer to this phenomenon as the “Non-Dominant Options Trade-Off effect”. Both the Numerosity effect and the Non-Dominant Options Trade-Off effect are examined in detail in the following sections.

### Numerosity effect

Both experiments contained pairs of conditions where one condition featured dots numbers that were a fixed proportion of the dot numbers in the other condition. Prior work has shown that such manipulations lead to relatively matched accuracy but higher confidence for the condition with a higher number of dots (Sepulveda et al., 2020). Here we replicated these previous results in both of our experiments. First, in Experiment 1, Conditions 1–3 were identical to Conditions 4–6, except that Conditions 4–6 had fewer dots (specifically, 80% of the dot numbers in Conditions 1–3). Correspondingly, Conditions 1–3 exhibited slightly higher accuracy (t(24) = 2.38, p = .03, Cohen’s d = .48) but much higher confidence than Conditions 4–6 (t(24) = 7.81, p = 4.8 X 10^−8^, Cohen’s d = 1.56). While both effects were significant, the effect size (Cohen’s d) for confidence was over three times larger than the effect size for accuracy. Second, in Experiment 2, Conditions 1–6 were identical to Conditions 7–12, except that Conditions 7–12 had fewer dots (specifically, 85.7% of the dot numbers in Conditions 1–6). These two groups of conditions did not significantly differ in accuracy (t(14) = 1.72, p = .11, Cohen’s d = .44) but Conditions 1–6 exhibited much higher confidence than Conditions 7–12 (t(14) = 6.11, p = 2.7 X 10^−5^, Cohen’s d = 1.58). The effect size for confidence was again over three times larger than the effect size for accuracy. These results show the presence of a robust Numerosity effect, where larger dot numbers lead to higher confidence but have only a small effect on accuracy.

### Non-Dominant Options Trade-Off effect

In Experiment 2, we manipulated the number of dots in the second and third most numerous colors to examine trade-offs between the two least probable options. Specifically, we explored how changes in the number of dots in these choices affect both confidence and accuracy. For instance, Conditions 3 and 5 had the same total numbers of dots, except that they were distributed differently among the three options: [98, 84, 48] in Condition 3 and [98, 72, 60] in Condition 5. Thus, the two conditions had equal number of dots for the top choice but the remaining dots were distributed more or less unevenly (84 and 48 in Condition 3; 72 and 60 in Condition 5). Direct comparison between these two conditions revealed that Condition 5 (more even distribution between the non-dominant options) was associated with much higher accuracy (t(14) = 9.92, p = 1.0 X 10^−7^, Cohen’s d = 2.56), which makes sense given that both of the non-dominant options became unlikely to be chosen over the correct answer. However, despite the vast difference in accuracy, confidence was matched between Conditions 3 and 5 (t(14) = 0.94, p = .36, Cohen’s d = .24), thus revealing a sizeable confidence-accuracy dissociation. We further replicated these findings when comparing Conditions 9 and 11 (which had [84, 72, 42] vs. [84, 62, 52] dots), such that Condition 11 produced much higher accuracy than Condition 9 (t(14) = 4.51, p = 4.9 X 10^−4^, Cohen’s d = 1.16), but the two conditions did not differ in confidence (t(14) = .94, p = .36, Cohen’s d = .24).

The Non-Dominant Options Trade-Off effect was even more pronounced when examining Conditions 3 and 4 ([98, 84, 48] vs. [98, 72, 72] dots). These conditions also featured a trade-off between the non-dominant options, but Condition 4 had a higher total number of dots, while still featuring fewer dots in the second most likely option. We found that Condition 4 featured significantly higher accuracy (t(14) = 2.56, p = .023, Cohen’s d = .66), but lower confidence (t(14) = −3.63, p = .003, Cohen’s d = −.94), thus revealing an even stronger confidence-accuracy dissociation. We replicated these results when comparing Conditions 9 and 10 (which had [84, 72, 42] vs. [84, 62, 62] dots), such that Condition 10 produced higher accuracy (t(14) = 3.05, p = .0087, Cohen’s d = .79) but lower confidence (t(14) = −2.94, p = .011, Cohen’s d = −.76). Overall, these results demonstrate the existence of a robust “Non-Dominant Options Trade-Off effect”, where a more even distribution between the non-dominant options tends to increase accuracy but decrease confidence.

### Developing models of the internal activations in our task

Having established the numerosity and Non-Dominant Options Trade-Off effects, we then sought to examine models of confidence that can reproduce these effects. However, before we can build a model of confidence, we need a model of the internal activations produced in our task and how they lead to the initial perceptual decision. Standard experimental manipulations (e.g., contrast for Gabor patches and coherence for random dot kinematograms) often need a separate parameter for each condition to describe the internal evidence distributions even for simple 2-choice tasks. In contrast, here we sought to build a very simple decision model where a single parameter can be used to model all conditions in each experiment (6 conditions in Experiment 1; 12 conditions in Experiment 2).

**Figure 3.**
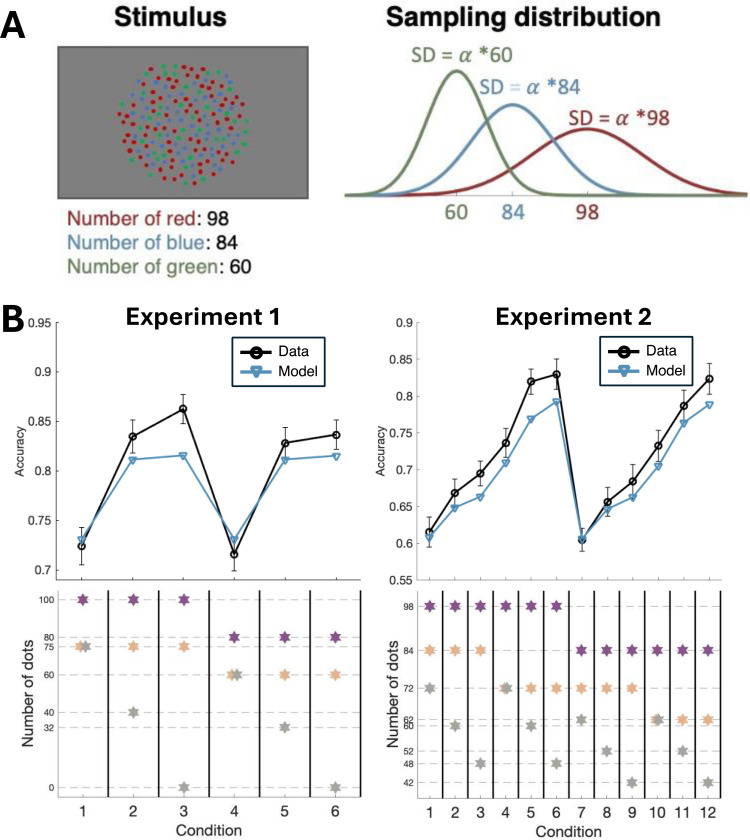
Modeling internal activation and decision. (A) The stimulus is a cloud of dots with different colors and we used the condition 2 in Experiment 2 as an example here. We modeled the internal activations for each color as a Gaussian distribution with a mean that is equal to the number of dots of that color and standard deviation that is equal to the number of dots of that color times a fixed parameter alpha (α). On a given trial, the activations for the three colors are obtained by independently sampling from the three distributions. (B) Model fit for the 1-parameter model (blue) overlaid on the empirical data (black). Despite its extreme simplicity, the 1-parameter model fits the empirical choice data well. Error bars depict SEM.

Specifically, we modeled the internal activation produced by n dots as a random variable, $X_{n}$, which follows a Gaussian distribution, such that $X_{n}∼N\left(\mu_{n},\sigma_{n}^{2}\right)$ where $\mu_{n}=n$ and $\sigma_{n}=\alpha n$ for some free parameter α (alpha). This model includes three components. First, we model the internal activations as Gaussian distributions. In reality, the distributions may be slightly skewed to the right, but this skew is likely small for large n’s, which is the situation we explore in the current experiments. Second, the model postulates that n dots give rise to an internal distribution centered on n, such that $\mu_{n}=n$. Third, the model postulates that the standard deviation of the Gaussian distribution is a linear function of the number of dots, such that $\sigma_{n}=\alpha n$. The reasoning behind the last two components of the model is that the activation produced by a set of $n_{1}+n_{2}$ dots should equal the sum of activations produced by a set of $n_{1}$ dots and a separate set of $n_{2}$ dots. Using this assumption, it follows that both the mean and the variance of the random $X_{n}$ are additive, which in turn directly leads to the above equations (see Methods). According to the model, larger numbers of dots produce internal activations that are both shifted to the right (i.e., have higher means) but also include larger uncertainty (i.e., have higher standard deviations) (Beran et al., 2006; Foster, 1923; Ratcliff & McKoon, 2018; Testolin & McClelland, 2021; Xiang et al., 2021). Critically, the model allows us to model the internal activations across a potentially unlimited number of conditions with a single parameter a (Figure 3A). Finally, in a k-choice experiment, the model assumes that people will choose the alternative that produces the highest internal activation.

To validate this approach, we examined how well this model could fit the experimental data and whether it could reproduce the decision patterns in our experiments. We found that despite its extreme simplicity, the decision model successfully reproduced the results from both experiments (Figure 3B). Qualitatively, in Experiment 1, accuracy increased as the number of dots from the least frequent color decreased (t(24) = −15.82, p = 3.37 X 10^−14^, Cohen’s d = −3.16). Similarly, in Experiment 2, accuracy generally increased as the number of dots from the two least frequent colors decreased (second choice: t(14) = −19.78, p = 1.25 X 10^−11^, Cohen’s d = −5.11, third choice: t(14) = −23.64, p = 1.10 X 10^−12^, Cohen’s d = −6.10). To quantitatively evaluate the model’s performance, we used the root mean squared error (RMSE) as a measure of goodness of fit. RMSE is a measure that quantifies the difference between the values predicted by the model and the values observed in the data, with lower values indicating a better fit. In our experiments, the RMSE values were .16 for Experiment 1 and .19 for Experiment 2, indicating a good fit to the data. We further examined the fitted alpha value (the free parameter in our model), which represents the noise level in one’s perceptual system. We found that the value was consistent across the two experiments despite the large differences in conditions between them: for Experiment 1 it was .27 (SD = .19), and for Experiment 2 it was also .27 (SD = .09). Note that the lower SD in Experiment 2 is likely due to the fact that this experiment had a lot more data per subject and thus led to less noisy alpha estimates. This consistency suggests that our model captured a stable aspect of perceptual processing across different experiments and supports the validity of our model.

While the 1-parameter model performed well, it cannot account for color-specific response biases, where individual subjects may be biased towards choosing one color more frequently. To address this limitation, we developed a slightly more complex, 4-parameter model. Specifically, we added two parameters that can account for color biases (see Methods) and also an additional parameter for lapse rates (Adler & Ma, 2018; Aitchison et al., 2015; Boundy-Singer et al., 2023; Denison et al., 2018). Due to its ability to capture color biases, the 4-parameter model demonstrated superior fit than the basic 1-parameter model, with average AIC reductions of 67.07 in Experiment 1 (3.55 × 10^14^ times more likely) and 230.89 in Experiment 2 (1.37 × 10^50^ times more likely). Further, the 4-parameter model again yielded consistent values for the fitted parameters across the two experiments. Specifically, the lapse rates were .063 (SD = .039) in Experiment 1 and .068 (SD = .078) in Experiment 2. Because the lapse rate parameter accounted for many of the observed errors, the fitted alpha values decreased compared to the 1-parameter model but remained consistent across the two experiments: .17 (SD = .047) for Experiment 1 and .18 (SD = .038) for Experiment 2. These results indicate that while the one-parameter model performs well, the 4-parameter model provides a more comprehensive account of the data, including subject-specific color biases.

### Comparing models of confidence computation

#### Confidence models

Having built a simple decision model that allows us to describe the internal activations in our task using very few parameters, we turned to the central problem of examining how confidence is computed in multi-alternative tasks. We evaluated three prominent models of confidence that have been proposed in the literature: the Top-2 Difference model (Top2Diff), which postulates that confidence reflects the difference in evidence for the top two options (Li & Ma, 2020; Shekhar & Rahnev, 2024b); the Bayesian Confidence Hypothesis (BCH), which postulates that confidence reflects the probability that the perceptual decision is correct (Hangya et al., 2016; Kepecs & Mainen, 2012; Meyniel et al., 2015; Pouget et al., 2016); and the Positive Evidence model (PE), which postulates that confidence reflects the strength of evidence for the chosen option only (Koizumi et al., 2015; Maniscalco et al., 2016; Peters et al., 2017; Samaha et al., 2019; Samaha & Denison, 2022).

**Figure 4.**
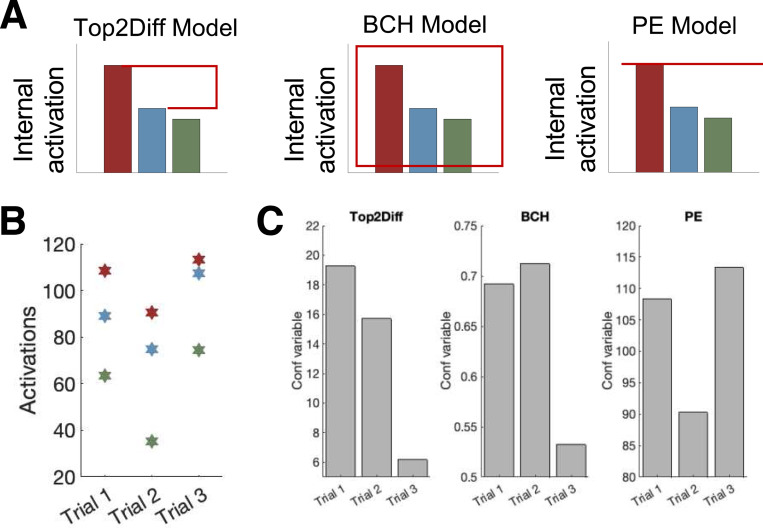
Confidence models. (A) Graphical depiction of the computations assumed by each model. The Top-2 Difference model (Top2Diff) computes confidence based on the difference in evidence for the top two options. The Bayesian Confidence Hypothesis (BCH) computes confidence based on the probability that the perceptual decision is correct. The Positive Evidence model (PE) computes confidence based on the strength of the evidence for the chosen option only. (B) Internal activations for red, blue, and green in three example trials. (C) The value of the confidence variable according to each model for the three trials in panel B. Top2Diff, BCH, and PE predict the highest confidence for trials 1, 2, and 3, respectively. Note that the confidence variables cannot be meaningfully compared across models.

These models lead to divergent predictions about confidence in our task. Indeed, Top2Diff predicts that confidence should be low when the difference of the top-2 activations is small, BCH predicts that confidence should be low when the posterior probabilities across choices are relatively uniform, and PE predicts that confidence should be low when the evidence for the chosen option is weak. While these conditions sometimes occur together, they often do not. For example, consider the three example trials in Figure 4A. For these three specific trials, Top2Diff predicts the highest confidence for trial 1, BCH predicts the highest confidence for trial 2, and PE predicts the highest confidence for trial 3 (Figure 4B). Thus, our task allows us to clearly dissociate between the three models.

#### Model comparison results

The divergent predictions of the Top2Diff, BCH, and PE models show that they can be distinguished via model fitting. Consequently, we fit the three model to the confidence data using the best-fitted parameters from the 4-parameter decision model, thus ensuring a common set of assumptions about the underlying distributions of sensory evidence among the confidence models. Therefore, fitting Top2Diff, BCH, and PE involved the simple process of fitting three parameters for the confidence criteria that would transform the continuous confidence variable in a confidence rating on a 4-point scale. We then used the Akaike Information Criterion (AIC) to determine how well each model fit the empirical data. Note that because all three models have the same number of free parameters, other metrics, such as the Bayesian Information Criterion, produce results identical to what is obtained using AIC.

**Figure 5.**
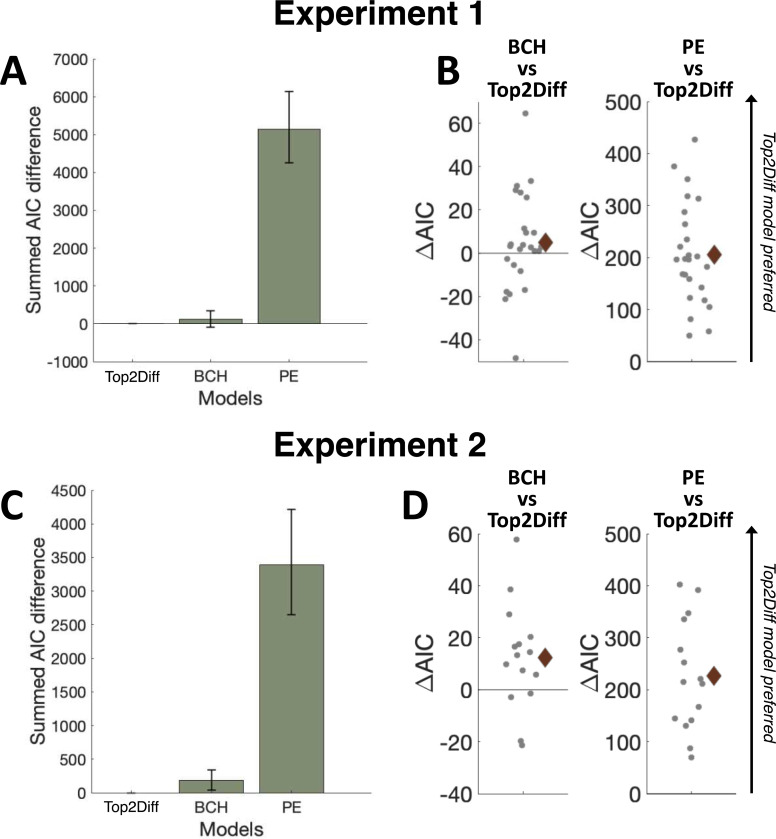
Model fitting results. (A) Summed AIC difference scores between each model and the best fitting model in Expt1. The error bar shows the 95% bootstrapped confidence interval. The Top2Diff model provided better fits compared to the BCH and the PE models. (B) The AIC difference between the BCH or the PE and the Top2Diff model for individual subjects in Expt 1. A positive value indicates that the Top2Diff model is preferred. The Top2Diff model outperformed the BCH model for 17 out of 25 subjects and outperformed the PE model for all 25 subjects. The brown diamond shows the mean value of the AIC differences across subjects. (C) Summed AIC difference scores between each model and the best fitting model in Expt 2. The Diff model significantly outperformed both the BCH and the Top2Diff model. (D). The AIC difference between the BCH or the PE and the Diff model for individual subjects in Expt2. The Top2Diff model outperformed the BCH model for 11 out of 15 subjects and outperformed the PE model for all 15 subjects.

We found compelling evidence in favor of the Top2Diff model in both experiments. In Experiment 1, Top2Diff outperformed BCH by a total of 123.72 summed AIC points (average of 4.95 per subject), indicating that the Top2Diff model is 7.33 X 10^26^ times more likely than BCH in the group (11.87 times more likely in the average subject; Figure 5A). More strikingly, Top2Diff outperformed PE by a total of 5.14 X 10^3^ summed AIC points (average of 205.57 per subject), indicating that the Top2Diff model is almost infinite times more likely than PE in the group (4.35 X 10^44^ times more likely in the average subject; Figure 5A). At the individual level, Top2Diff outperformed BCH for 17 out of 25 subjects and the PE model for all 25 subjects (Figure 5B). Experiment 2 featured 2.5 times more trials per subject than Experiment 1 (1440 vs. 576), thus allowing for even more stable results as the level of the individual. Indeed, we found that in Experiment 2, Top2Diff outperformed BCH by a total of 184.70 AIC points (average of 12.31 per subject), indicating that the Top2Diff model is 1.28 X 10^40^ more likely than BCH in the group (471.8 times more likely in the average subject; Figure 5C). More strikingly, Top2Diff outperformed PE by a total of 3.39 × 10^3^ AIC points (average of 225.18 per subject), indicating that the Top2Diff model is again almost infinite times more likely than PE in the group (1.30 × 10^49^ times more likely in the average subject; Figure 5C). At the individual subject level, the Top2Diff model outperformed the BCH model for 11 out of 15 subjects and outperformed the PE model for all 15 subjects (Figure 5D). These results provide robust evidence that the Top2Diff model offers the best account of confidence computation in our multi-alternative perceptual decision-making tasks.

#### Qualitative fits to the confidence data

**Figure 6.**
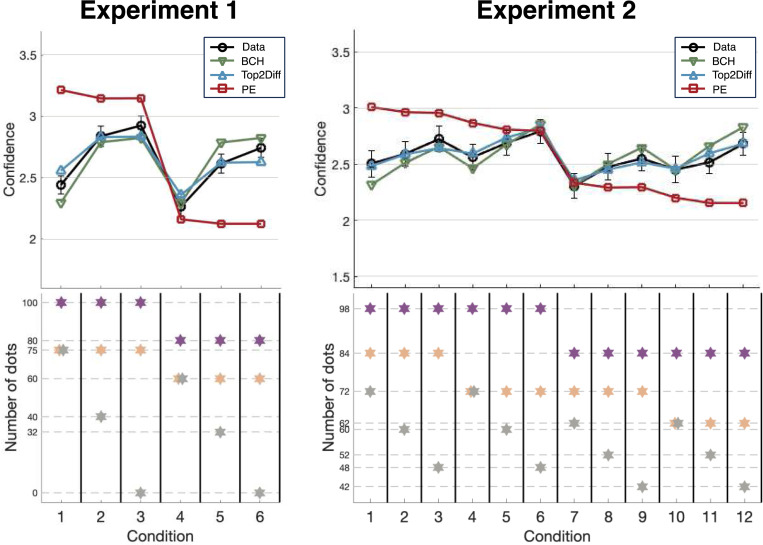
Confidence models fits. The predicted confidence for each of the three models – Top2Diff, BCH, and PE – is plotted against their observed values. The black lines show the observed values, and the colored lines show the predicted values for each model. For both Experiments 1 and 2, the Top2Diff model provides the best fit to the data, with the BCH model providing an almost equally good fit. In contrast, the PE model completely failed to capture the pattern of the observed confidence data. Error bars show SEM.

Having assessed the quantitative model fits for Top2Diff, BCH, and PE, we examined each model’s ability to provide a good qualitative fit to the confidence data (Figure 6). Specifically, we tested the ability of each of the models to correctly reproduce the size of the Numerosity and Non-Dominant Options Trade-Off effects.

We found that Top2Diff demonstrated the best overall fit to the empirical data. It successfully reproduced the pattern of increased confidence as the number of dots from the least frequent colors decreased (all p’s < .05 across both experiments). Critically, Top2Diff also accurately reproduced the Numerosity effect where conditions with higher numbers of dots but equal ratios of dot numbers lead to higher confidence. Indeed, Top2Diff predicted the correct magnitude of confidence increase for conditions with higher numbers of dots. Specifically, there were no significant differences in confidence between empirical effects and model predictions between Conditions 1–3 and Conditions 4–6 in Experiment 1 (empirical difference = .19, model difference = .20; t(24) = −.45, p = .66, Cohen’s d = −.090) and between Conditions 1–6 and Conditions 7–12 in Experiment 2 (empirical difference = .15, model difference = .13; t(24) = .62, p = .54, Cohen’s d = .16). The Top2Diff model also partially captured the Non-Dominant Options Trade-Off effect in Experiment 2 where a more even distribution of evidence among non-dominant options tends to increase accuracy but decreases or maintains confidence. For pairs of conditions where the first condition exhibits higher confidence but lower accuracy than the second condition (i.e., Conditions 3 vs. 4 and 9 vs. 10), the model successfully predicted higher confidence though with slightly smaller magnitude compared with empirical data (Conditions 3 vs. 4: empirical difference = .16, model difference = .05; t(14) = 2.60, p = .021, Cohen’s d = .67; Conditions 9 vs. 10: empirical difference = .10, model difference = .06; t(14) = .80, p = .44, Cohen’s d = .21). In pairs of conditions where the first condition exhibits lower accuracy but matched confidence to the second condition (i.e., Conditions 3 vs. 5 and 9 vs. 11), the model predicted similar confidence levels while capturing accuracy differences, though there were again slight deviations from the empirical data (Conditions 3 vs. 5: empirical difference = −.03, model difference = .09, t(14) = 3.91, p = .0016, Cohen’s d = 1.01; Conditions 9 vs. 11: empirical difference = −.03, model difference = .08, t(14) = 2.62, p = .02, Cohen’s d = .68). Overall, these results indicate that Top2Diff provides a reasonable approximation of the data by successfully reproducing the Numerosity effect and capturing key patterns of the Non-Dominant Options Trade-Off effect, with only minor deviations in predicting confidence differences between specific conditions.

The BCH model provided an overall good fit to the empirical confidence ratings but failed to capture all qualitative effects. Like Top2Diff, it successfully reproduced the pattern of increased confidence as the number of dots from the least frequent colors decreased (all p’s < .05 across both experiments). However, the BCH model failed to reproduce the Numerosity effect. Indeed, BCH predicted no difference in confidence between Conditions 4–6 and Conditions 1–3 in Experiment 1 (means difference = .00), whereas the empirical data showed a difference of .19 (t(24) = 7.88, p = 4.14 X 10^−8^, Cohen’s d = 1.58). Similarly, BCH failed to reproduce the Numerosity effect between Conditions 1–6 and Conditions 7–12 in Experiment 2 (means difference = 0.01), whereas the empirical data showed a difference of 0.15 (t(14) = 5.7, p = 5.48 X 10^−5^, Cohen’s d = 1.47). The small difference of .01 that BCH predicts in Experiment 2 reflects minor ratio differences between Conditions 1–6 and 7–12 that were unavoidable due to experimental constraints (see Methods for more details). However, despite its poor performance on the Numerosity effect, BCH captured the Non-Dominant Options Trade-Off effect in Experiment 2. For pairs of conditions where the first condition exhibits higher confidence but lower accuracy than the second condition (i.e., Conditions 3 vs. 4 and 9 vs. 10), the model successfully predicted higher confidence with slightly larger magnitude compared with empirical data (Conditions 3 vs. 4: empirical difference = .16, model difference = .19; t(14) = .75, p = .46, Cohen’s d = .19; Conditions 9 vs. 10: empirical difference = .10, model difference = .20; t(14) = 1.86, p = .084, Cohen’s d = .48). In pairs of conditions where the first condition exhibits lower accuracy but matched confidence to the second condition (i.e., Conditions 3 vs. 5 and 9 vs. 11), the model predicted similar confidence levels while capturing accuracy differences, closely matching the empirical data (Conditions 3 vs. 5: empirical difference = −.03, model difference = −.02, t(14) = 1.66, p = .12, Cohen’s d = .43; Conditions 9 vs. 11: empirical difference = −.03, model difference = −.02, t(14) = 1.08, p = .30, Cohen’s d = .28). Overall, while BCH captured the Non-Dominant Options Trade-Off effect, its inability to reproduce the Numerosity effect suggests some limitations in accounting for all patterns in the behavioral data.

Unlike Top2Diff and BCH, the PE model completely failed to capture the confidence data pattern in both Experiments 1 and 2. In Experiment 1, while empirical confidence increased as the least frequent color decreased, the PE model predicted the opposite effect (t(24) = 8.29, p = 1.65 X 10^−8^, Cohen’s d = 1.66). In Experiment 2, PE failed to capture the increase in confidence as the two least frequent colors decreased and instead predicted a decrease in confidence as the two least frequent colors decreased (second choice: t(14) = 11.84, p = 1.11 X 10^−8^, Cohen’s d = 3.06; third choice: t(14) = 2.68, p = .018, Cohen’s d = .69). As can be expected (see Methods), the PE model exhibited a strong Numerosity effect in both experiments, but the strength of the effect much exceeded what was observed in the empirical data (Experiment 1: empirical difference = .19, predicted difference = 1.03, t(24) = 16.96, p = 7.23 X 10^−15^, Cohen’s d = 3.39; Experiment 2: empirical difference = .15, predicted difference = .55, t(14) = 14.49, p = 8.01 X 10^−10^, Cohen’s d = 3.74). Overall, PE showed the expected strong Numerosity effect but completely failed to describe the overall confidence data, including the size of the Numerosity effect.

## Discussion

Our study addresses a key challenge in understanding confidence computation in multi-alternative tasks: modeling how the brain transforms external sensory information into internal representations. Using a novel behavioral paradigm with a dot numerosity task, we demonstrated that this complex transformation can be captured by a remarkably simple one-parameter decision model – a significant advance over previous approaches that typically required numerous parameters. The success of our model across up to 12 different conditions in two experiments with multi-alternative choices demonstrated its robustness and generalizability. This modeling framework also provided a unique opportunity to compare three leading theories of confidence computation in multi-alternative tasks. We found that the Top-2 Difference (Top2Diff) model best explained the computation underlying human confidence data in tasks with multiple alternatives, challenging the two other prominent theories: the Bayesian Confidence Hypothesis (BCH) and the Positive Evidence (PE) model. Thus, our work not only establishes a powerful new experimental framework that can characterize internal representations across unlimited multi-alternative conditions with minimal assumptions but also challenges two prominent theories of confidence computation.

Our work addresses two fundamental challenges in perceptual decision-making. First, one of the limiting factors for understanding and modeling perceptual decisions is knowing how external sensory information is transformed into internal representation (Abid, 2019; Denison et al., 2018; Odegaard et al., 2018; Rahnev et al., 2011). Traditional approaches using Gabor patches or random dot motion stimuli require multiple free parameters to model the transformation from stimulus properties to internal decision variables and stimulus manipulations sometimes permit modeling with divergent sets of parameters (Lee et al., 2023). Second, most studies in the field focus on 2-choice tasks, and building computational models for multi-alternative tasks remains rare (Li & Ma, 2020; Rahnev et al., 2022). Given the inherent complexity of modeling even binary choices, extending standard paradigms to multi-alternative tasks becomes computationally intractable (Shekhar & Rahnev, 2024a). Using our dot numerosity paradigm, here we address both challenges simultaneously. First, we establish a clear and simple mapping between external stimuli and internal representations using as little as one parameter, addressing the transformation problem. Second, this mapping naturally extends to situations with multiple alternatives, enabling the investigation of confidence in multi-alternative tasks while maintaining model parsimony.

The success of our decision model stems from its strong theoretical foundation. The model’s core assumption—that the standard deviation of internal activations increases linearly with stimulus magnitude—aligns with the well-established Weber’s law and its extensions in psychophysics (Foster, 1923; Gibbon, 1977; Gibbon & Church, 1981; Nieder & Miller, 2003; Petzschner & Glasauer, 2011; Roach et al., 2017; Treisman, 1964; Xiang et al., 2021). This principle, which suggests that response variability scales with stimulus magnitude, has been observed across various perceptual domains (Gibbon, 1977; Roach et al., 2017; Treisman, 1964; Xiang et al., 2021), neuronal responses (Dean, 1981; Dosher & Lu, 1999, 2017; Lu & Dosher, 2008; Tolhurst et al., 1983), and even metacognitive noise (Shekhar & Rahnev, 2021). Thus, employing a dot numerosity task allows us to establish a precise mapping between stimulus manipulation and internal representations in multi-alternative tasks. Empirically, the success of the model can be seen not only in the good overall fits but also in the fact that the model’s key parameter, alpha, which quantifies the noise level in the perceptual system, demonstrates remarkable consistency between Experiments 1 and 2. The strong theoretical foundation, coupled with the empirical success of our single-parameter models, underscore the promise of dot numerosity tasks in perceptual decision-making research.

Our results showed the Top2Diff model as providing the best fit to the confidence data. This success aligns with recent findings suggesting that confidence computations rely on relative evidence comparisons (Li & Ma, 2020; Shekhar & Rahnev, 2024a). The Top2Diff model has also received some indirect support from the success of SDT- and evidence-accumulation-based models (Hellmann et al., 2022; Maniscalco & Lau, 2016; Pleskac & Busemeyer, 2010; Ratcliff & Starns, 2009; Shekhar & Rahnev, 2024a), as these models typically compute confidence based on the difference between evidence supporting competing alternatives. Top2Diff offers a straightforward approach to computing confidence in multi-alternative tasks by focusing on the difference between the top two options. The model achieves computational simplicity through a single subtraction operation between the two highest activations, but it also maintains a high degree of informativeness by capturing the most relevant information for confidence computation. Empirically, Top2Diff predicts the Numerosity effect because higher absolute dot numbers lead to higher absolute evidence differences in the top two choices even when the ratios are fixed, which leads to higher confidence ratings. It predicts the Non-Dominant Options Trade-Off effect because when the non-dominant options were sampled as the top-2 choices through random sampling, the conditions with more evenly distributed non-dominant choices led to a smaller absolute evidence difference between these two options, resulting in an overall lower confidence. Thus, the simple computation postulated by Top2Diff is consistent with prior modeling work, achieves high degree of informativeness, and reproduces key empirical patterns in our multi-alternative task.

In stark contrast to Top2Diff, the PE model completely failed to fit the confidence data. The PE model was proposed as a means to explain counterintuitive findings where greater stimulus energy leads to higher confidence even if there is no change in accuracy (Koizumi et al., 2015; Maniscalco et al., 2016, 2021; Peters et al., 2017; Samaha & Denison, 2022). However, in the context of the dot numerosity task, PE makes predictions that are both counterintuitive and empirically false. For example, PE predicts higher confidence in conditions where the non-dominant option has a higher number of dots (e.g., confidence would be higher for [100,90] than for [100,70]). This prediction occurs because on trials where random sampling leads to the second option being chosen, conditions with a higher number of dots in the second option will produce higher confidence ratings. This causes the average confidence across all trials to be higher with higher second options, a prediction that contradicts both intuition and empirical findings. In the extreme case, PE predicts higher confidence for situations with two equal evidence options (e.g., [100, 100]) compared to situations with one clearly dominant option (e.g., [100, 0]) – a prediction that, although not explicitly tested here, is clearly nonsensical in the context of the dot numerosity task. The strong limitations of the PE model in the dot numerosity task are mirrored in recent work that has uniformly failed to support the PE computation when directly compared against alternatives (Shekhar & Rahnev, 2024a, 2024b; Webb et al., 2023). Our results thus add to a growing literature suggesting that confidence computations do not only consider the evidence for the chosen alternative.

While the BCH model performed much better than PE, it failed to capture the human confidence data as well as Top2Diff. Most notably, BCH failed to capture the Numerosity effect, where conditions with higher absolute dot numbers produce greater confidence despite matched accuracy. Indeed, from BCH’s perspective, conditions with identical ratios of dots (e.g., [100,75] vs [80,60]) should produce identical confidence because they yield the same probability of being correct – the relative evidence between options remains constant regardless of absolute dot numbers. Our results contribute to a large body of evidence showing that BCH does not accurately capture human confidence (Adler & Ma, 2018; Li & Ma, 2020; Locke et al., 2022; Xue et al., 2023). In fact, BCH would appear a priori unlikely for complex, multi-alternative tasks like the ones here. Indeed, computing the exact probabilities of being correct in our task requires integrating over multiple possible outcomes (see Methods) – a computationally demanding process that would be difficult for the brain to implement. Together, empirical findings and theoretical considerations suggest that confidence judgments likely rely on simpler computations that do not always accurately estimate the probability of being correct.

Our results revealed two behavioral effects that lead to confidence-accuracy dissociations. The first is the well-documented Numerosity effect (Sepulveda et al., 2020), where confidence is higher in conditions with a greater overall number of dots even when the ratio of dot numbers (and actual accuracy) are matched. The second is our novel discovery of the Non-Dominant Options Trade-Off effect, where a more even distribution of the non-dominant options leads to increased accuracy but decreased or matched confidence. These effects produce robust confidence-accuracy dissociations and can serve as qualitative signatures that can help constrain theories of metacognition as any viable model of confidence must be able to account for both of these effects.

In conclusion, our study establishes a novel behavioral paradigm that addresses two fundamental challenges in perceptual decision making. First, we demonstrate that internal representations in multi-alternative tasks can be precisely described using a simple, one-parameter model, enabling us to fit data from potentially unlimited number of conditions. Second, using this paradigm, we tested three prominent theories of confidence computation and found strong evidence that confidence reflects the difference between the top two alternatives (Top2Diff model).

## Methods

### Subjects

Experiment 1 featured 25 subjects (12 female; mean age = 20.0 years) each completing a total of 576 trials in single session. Experiment 2 featured 15 subjects (8 female; mean age = 19.3 years) who completed 1,440 trials each over two sessions. All subjects had normal or corrected to normal vision and signed informed consent. Experimental procedures were approved by the Georgia Institute of Technology Institutional Review Board.

### Experimental design

Each trial in both experiments began with a fixation point at the center of the screen for a duration of 500 ms, followed by the presentation of the stimulus for 500 ms (Figure 1A). The stimulus was a cloud of dots with a radius of 8 degrees of visual angle (231 pixels) consisting of either two or three different colors: red (RGB: 255, 32, 32), green (RGB: 0, 180, 0), and blue (RGB: 15, 15, 255). Individual dots had a diameter of 5 pixels. After the presentation of the stimulus, a response screen was presented until the subjects provided a decision. The subjects’ task was to indicate which was the dominant color (i.e., the color with the most dots) in the cloud of dots using keyboard buttons ‘Z’, ‘X’, and ‘C’ for red, green, and blue respectively. After the subjects gave their response, a confidence screen appeared and was presented until the subjects made a confident response. The confidence ratings were on a scale of one to four. Subjects indicated their decision as well as confidence level using the keyboard.

In Experiment 1, participants completed the experiment in a single session consisting of 3 runs with 4 blocks per run, with each block containing 48 trials, totaling 576 trials per subject. Experiment 2 was completed over two sessions. Each session consisted of 3 runs with 5 blocks per run, and each block contained 48 trials. In total, subjects in Experiment 2 completed 1,440 trials across the two sessions. In both experiments, a one-second break was provided between trials, with 15-second breaks between blocks. Between runs, subjects could take breaks of unlimited duration and continue the experiment by pressing any key when ready. We ensured that each color appeared equally often in each position (dominant, middle, and fewest dots). To do so, the relative positions of the three colors were counterbalanced across trials using six different possible configurations: [red, blue, green], [red, green, blue], [blue, red, green], [blue, green, red], [green, red, blue], and [green, blue, red]. The dot numbers for each condition are illustrated in Figure 3 and specified in Figure 3B and in Table 1.

In Experiment 1, Conditions 1–3 and 4–6 were perfectly matched in their ratios, with Conditions 4–6 using exactly 80% of the dot numbers in Conditions 1–3. For instance, in Conditions 1 vs. 4, the ratios are identical: 100:75:75 vs. 80:60:60, both yielding 0.75 for the ratio between second-highest and highest dots. This perfect matching was maintained across all paired conditions (2 vs. 5: 100:75:40 vs. 80:60:32, and 3 vs. 6: 100:75:0 vs. 80:60:0).

In Experiment 2, Conditions 1–6 and 7–12 were designed to maintain similar evidence ratios while using different absolute numbers (Conditions 7–12 used approximately 85.7% of the dot numbers in Conditions 1–6). However, due to the constraint that dot numbers must be whole integers, small deviations in ratios were unavoidable. For example, in Condition 1, the ratios between the three dot numbers were 1, .86, and .73. Condition 7 was designed to have very similar ratios and showed nearly identical proportions of 1, .86, and .74, with only a small deviation in the lowest ratio. Similar small deviations exist across other matched conditions (Table 1).

Before starting each experiment, subjects completed four training blocks. The first training block contained 15 trials with a stimulus presentation time of three seconds to allow familiarization with the stimulus. The second training block included 25 trials with 1.5-second stimulus presentation. The third and fourth training blocks each consisted of 25 trials with the same 500 ms presentation time as the main experiment. In Experiment 1, subjects were explicitly informed that they would see either two or three colors in each trial, while in Experiment 2, where all trials contained three colors, this instruction was omitted. Feedback about response accuracy was provided after each trial during the first three training blocks, while the fourth training block had no feedback, matching the conditions of the main experiment. During training, subjects were instructed to use the whole confidence scale and to maintain fixation on the central fixation point throughout each trial.

### Decision model

The main goal of our decision model was to describe how the brain transforms the numbers of dots presented into internal sensory evidence on which the decisions are based. Let $X_{n}$ be a random variable that represents the internal activation produced by n dots. Following the long tradition of modeling sensory evidence in perceptual decision making (Green & Swets, 1966), we assume $X_{n}$ follows a Gaussian distribution: $X_{n}∼N\left(\mu_{n},\sigma_{n}^{2}\right)$, where $\mu_{n}$ is the mean and $\sigma_{n}$ is the standard deviation of the distribution. Since the activation produced by $n_{1}+n_{2}$ dots should be equal to the sum of activations produced by $n_{1}$ and $n_{2}$ dots separately, this means that $X_{n_{1}+n_{2}}=X_{n_{1}}+X_{n_{2}}$, which in turn leads to the following equations:

$$\mu_{n_{1}+n_{2}}=\mu_{n_{1}}+\mu_{n_{2}}$$

and

$$\sigma_{n_{1}+n_{2}}^{2}=Var\left(X_{n_{1}+n_{2}}\right)=Var\left(X_{n_{1}}\right)+Var\left(X_{n_{2}}\right)+2\mathrm{Cov}\left(X_{n_{1}},X_{n_{2}}\right)\;\;=\sigma_{n_{1}}^{2}+\sigma_{n_{2}}^{2}+2\rho_{X_{n_{1}},X_{n_{2}}}\sigma_{n_{1}}\sigma_{n_{2}}$$

Because any random variability in the combined activation $X_{n_{1}+n_{2}}$ must be perfectly shared between $X_{n_{1}}$ and $X_{n_{2}}$ (as they contribute to the same total), the correlation coefficient $\rho_{X_{n_{1}},X_{n_{2}}}$ between $X_{n_{1}}$ and $X_{n_{2}}$ equals 1. Therefore:

$$\sigma_{n_{1}+n_{2}}^{2}=\sigma_{n_{1}}^{2}+\sigma_{n_{2}}^{2}+2\sigma_{n_{1}}\sigma_{n_{2}}={\left(\sigma_{n_{1}}+\sigma_{n_{2}}\right)}^{2}$$

which simplifies to:

$$\sigma_{n_{1}+n_{2}}=\sigma_{n_{1}}+\sigma_{n_{2}}$$

In other words, both the mean and the standard deviation of the random variable $X_{n}$ are additive. Given that presenting zero dots should produce zero activation $\left(\mu_{0}=0\right)$ and zero variability $\left(\sigma_{0}=0\right)$, it follows that both the mean and the standard deviations are linear functions of the number of dots. However, since a multiplicative change in activations makes no difference to the relative evidence across different colors, without loss of generality, we can assume that $\mu_{n}=n$ and $\sigma_{n}=an$ for some free parameter 𝑎 that represents the noise level in the subject’s perceptual system. This parameterization allows us to model sensory activations across all conditions with a single parameter a (Figure 3A). Finally, when several colors are presented, the model assumes that subjects choose the color that produces the highest activation on each trial.

To account for individual differences in color preferences, we developed a 4-parameter extension of the basic 1-parameter model introduced above. According to this model, the internal activation for each color is multiplied by a color-specific factor: $m_{r}$ for red, $m_{g}$ for green, and $m_{b}$ for blue, such that $X_{n}∼N\left(mn,a^{2}m^{2}n^{2}\right)$, where m represents the color multiplier for individual color bias, and n represents the number of dots from each color. However, as above, because a multiplicative change in activations makes no difference to the relative evidence across different colors, without loss of generality, we can assume that $m_{r}=1$, which leads to only two free parameters ($m_{g}$ and $m_{b}$). A final parameter λ captures the lapse rate – the proportion of trials where subjects make random decisions unrelated to the stimulus (Adler & Ma, 2018; Aitchison et al., 2015; Boundy-Singer et al., 2023; Denison et al., 2018). Thus, the complete model has four free parameters: the noise parameter a, two color multipliers ($m_{g}$ and $m_{b}$), and the lapse rate λ.

### Confidence models

Having established the decision model parameters, we next examined three different models of confidence computation while keeping the decision parameters fixed. For all models, confidence ratings are generated by placing decision criteria on their respective confidence variables, with the location of these criteria determining how the continuous confidence variable is mapped to four discrete confidence ratings. Three free parameters corresponding to the three confidence criteria were used for all models. We compared three prominent hypotheses: the Top-2 Difference (Top2Diff) model, the Bayesian Confidence Hypothesis (BCH) model, and the Positive Evidence (PE) model. Below, we expand on the assumptions of each model and give equations for the confidence variable that each assumes.

### Top-2 Difference (Top2Diff) model

According to the Top2Diff model, confidence reflects the difference between evidence for the top two sensory signals (Li & Ma, 2020; Shekhar & Rahnev, 2024a). In the context of a 3-choice task, on a given trial, we may observe specific activation values $\left[x_{r},x_{g},x_{b}\right]$, corresponding to the activations for red, blue, and green. Then, the confidence variable, $C_{Top\text{2}Diff}$, is computed as:

$$c_{Top2Diff}\left(x_{r},x_{g},x_{b}\right)=\max\left(x_{r},x_{g},x_{b}\right)-\text{max2}\left(x_{r},x_{g},x_{b}\right)$$

where $\text{max}\left(x_{r},x_{g},x_{b}\right)$ is the highest value among the three, and $\text{max2}\left(x_{r},x_{g},x_{b}\right)$ is the second highest value.

The model predicts higher confidence when there is a larger difference between the top two options. Importantly, in our numerosity task, this means that conditions with higher absolute dot numbers should produce higher confidence (due to larger absolute differences between options) even when the ratio between options is matched.

### Bayesian Confidence Hypothesis (BCH) model

According to the BCH model, confidence reflects the probability of a certain decision being correct, representing a normative solution to confidence computation (Hangya et al., 2016; Kepecs & Mainen, 2012; Meyniel et al., 2015; Pouget et al., 2016). Let L(x) be the likelihood function that describes the likelihood that a given internal activation x is produced by different number of dots. As above, we assume that on a given trial, we observe specific activation values $\left[x_{r},x_{g},x_{b}\right]$, corresponding to the activations for red, blue, and green. Assuming the subject chooses red (which means that $x_{r}>x_{g}$ and $x_{r}>x_{b}$), the confidence variable would be the probability that red is indeed the correct answer. Computing this probability is computationally expensive and has no closed-form solution. In theory, the correct computation is to integrate over all possible tuples $\left(k_{r},k_{g},k_{b}\right)$ that represent the possible number of red, green, and blue dots. The values of k can be anywhere from 1 to infinity, but to make the computations tractable we restricted them to between 1 and 200. We then computed the probability of being correct using the formula:

$$c_{BCH}\left(x_{r},x_{g},x_{b}|x_{r}>x_{g},x_{r}>x_{b}\right)\;=\frac{L\left(k_{r}>k_{g},k_{r}>k_{b}\right)+\frac{1}{2}L\left(k_{r}=k_{g}>k_{b}\right)+\frac{1}{2}L\left(k_{r}=k_{b}>k_{g}\right)+\frac{1}{3}L\left(k_{r}=k_{b}=k_{g}\right)}{L\left(k_{r},k_{g},k_{b}\right)}$$

The numerator in the formula integrates over all cases where $k_{r}$, the possible number of the red dots, is greater than $k_{g}$ and $k_{b}$, the possible number of the green and blue dots. Note that if two of these numbers are equal, then the probability of being correct in that case is 50%, whereas if all three numbers are the same, the probability of being correct is 33.3%. In contrast, the denominator integrates over all possible values of $k_{r}$, $k_{g}$, and $k_{b}$.

The denominator in the equation above can be expanded to:

$$L\left(k_{r},k_{g},k_{b}\right)=\sum_{k_{r}=1}^{200}L_{k_{r}}\left(x_{r}\right)*\sum_{k_{g}=1}^{200}L_{k_{g}}\left(x_{g}\right)*\sum_{k_{b}=1}^{200}L_{k_{b}}\left(x_{b}\right)$$

where $L_{k}(x)$ is the function that describes the likelihood that internal activation x was produced by k dots. Based on the assumptions of the decision model, $L_{k}(x)=\frac{1}{\sqrt{2\text{Π}\alpha^{2}k^{2}}}e^{-\frac{{\left(x-k\right)}^{2}}{2\alpha^{2}k^{2}}}$. As for the numerator of the formula above, we have:

$$L(k_{r}>k_{g},k_{r}>k_{b})+\frac{1}{2}L\left(k_{r}=k_{g}>k_{b}\right)+\frac{1}{2}L\left(k_{r}=k_{b}>k_{g}\right)+\frac{1}{3}L\left(k_{r}=k_{b}=k_{g}\right)\;\;=\sum_{k_{r}=1}^{200}L_{k_{r}}\left(x_{r}\right)\left({\sum_{k_{g}=1}^{k_{r}-1}L}_{k_{g}}\left(x_{g}\right)+0.5L_{k_{r}}\left(x_{g}\right)\right)\left(\sum_{k_{b}=1}^{k_{r}-1}L_{k_{b}}\left(x_{b}\right)+0.5L_{k_{r}}\left(x_{b}\right)\right)\;\;+\frac{1}{12}\sum_{k_{r}=1}^{200}L_{k_{r}}\left(x_{r}\right)L_{k_{r}}\left(x_{g}\right)L_{k_{r}}\left(x_{b}\right)$$

Note that the last formula includes corrections for cases where $k_{r}=k_{g}>k_{b}$ (where red is assumed to be correct with 50% probability), $k_{r}=k_{b}>k_{g}$ (where red is assumed to be correct with 50% probability), and $k_{r}=k_{g}=k_{b}$ (where red is assumed to be correct with 33.3% probability).

The BCH model predicts that confidence should track only the probability of being correct. Therefore, conditions with matched accuracy should produce matched confidence regardless of absolute dot numbers, which is why BCH does not show the Numerosity effect.

### Positive Evidence (PE) model

The PE model proposes that confidence reflects only the strength of evidence supporting the chosen option, ignoring evidence for alternatives (Koizumi et al., 2015; Maniscalco et al., 2016; Peters et al., 2017; Samaha et al., 2019; Samaha & Denison, 2022). As above, we assume that on a given trial, we observe specific activation values $\left[x_{r},x_{g},x_{b}\right]$, corresponding to the activations for red, blue, and green. Then, the confidence variable $C_{PE}$ is simply:

$$c_{PE}\left(x_{r},x_{g},x_{b}\right)=\max\left(x_{r},x_{g},x_{b}\right)$$

In our task, PE predicts that confidence will increase whenever more dots are presented for any option, as this increases the maximum possible evidence value that can be sampled, regardless of the relative distribution of evidence between options.

#### Model fitting and model comparison

Model fitting was performed through a maximum likelihood estimation (MLE) strategy, aimed at identifying parameter sets that optimize log-likelihood for full probability distribution of responses, using established procedures from our lab (Rahnev et al., 2011; Rahnev et al., 2012; Shekhar & Rahnev, 2021). The calculation of log-likelihood, logL, was performed using the following formula:

$$logL=\sum_{i,j,k}\log\left(p_{ijkc}\right)*n_{ijk}$$

where $p_{ijk}$ represents the probability of a given response, $n_{ijk}$ denotes the count of trials in the empirical data, i represents the stimulus category (with i={1,2,3}), j represents the confidence response (with j={1,2,3,4}), and k represents the condition, k={1,2,…,6} for experiment 1 and k={1,2,…,12} for experiment 2, and c represents color configuration (c={1,…,6}, corresponding to the six possible arrangements of the three colors: [red, blue, green], [red, green, blue], [blue, red, green], [blue, green, red], [green, red, blue], and [green, blue, red]). The parameter search was conducted using the Bayesian Adaptive Direct Search (BADS) toolbox, version 1.0.5 (Acerbi & Ma, 2017). To validate the robustness of model fits, we ran the fitting algorithm twice for each model and selected the fitted parameters associated with the highest log-likelihood values.

Model performance was assessed through the Akaike Information Criterion (AIC), which evaluates how well a model replicates the observed data, adjusting for the complexity added by additional parameters. AIC was computed using the standard formula: AIC=−2logL+2k, where k is the count of free parameters in the model and indicates the trial number (note that k=3 for all three confidence models examined above). Models with lower AIC values are considered to offer a superior fit. To determine the statistical significance in AIC comparisons, we generated bootstrapped 95% confidence intervals for these differences, aggregating data from all participants. These intervals were derived from 100,000 data samples, with intervals excluding zero indicating significant AIC differences between models.

#### Data and code

Data and code for analysis and model fitting for both experiments are available at https://osf.io/sr9j4/.

## Figures and Tables

**Table 1.** Number of dots in each condition for each experiment

Condition	Dot numbers (Expt 1)	Ratios (Expt 1)	Dot numbers (Expt 2)	Ratios (Expt 2)
Cond 1	100, 75, 75	1, .75, .75	98, 84, 72	1, .86, .73
Cond 2	100, 75, 40	1, .75, .40	98, 84, 60	1, .86, .61
Cond 3	100, 75, 0	1, .75, 0	98, 84, 48	1, .86, .49
Cond 4	80, 60, 60	1, .75, .75	98, 72, 72	1, .73, .73
Cond 5	80, 60, 32	1, .75, .40	98, 72, 60	1, .73, .61
Cond 6	80, 60, 0	1, .75, 0	98, 72, 48	1, .73, .49
Cond 7			84, 72, 62	1, .86, .74
Cond 8			84, 72, 52	1, .86, .62
Cond 9			84, 72, 42	1, .86, .50
Cond 10			84, 62, 62	1, .74, .74
Cond 11			84, 62, 52	1, .74, .62
Cond 12			84, 62, 42	1, .74, .50
